# Transfersome-Based Delivery of Optimized Black Tea Extract for the Prevention of UVB-Induced Skin Damage

**DOI:** 10.3390/pharmaceutics17080952

**Published:** 2025-07-23

**Authors:** Nadia Benedetto, Maria Ponticelli, Ludovica Lela, Emanuele Rosa, Flavia Carriero, Immacolata Faraone, Carla Caddeo, Luigi Milella, Antonio Vassallo

**Affiliations:** 1 Department of Health Sciences, University of Basilicata, Via dell’Ateneo Lucano 10, 85100 Potenza, Italy; nadia.benedetto@unibas.it (N.B.); maria.ponticelli@unibas.it (M.P.); ludovica.lela@unibas.it (L.L.); flavia.carriero@unibas.it (F.C.); luigi.milella@unibas.it (L.M.); antonio.vassallo@unibas.it (A.V.); 2 Department of Pharmacy, University of Studi di Salerno, Via Giovanni Paolo II n. 132, 84084 Fisciano, Italy; erosa@unisa.it; 3 Innovative Startup Farmis s.r.l., Via Nicola Vaccaro 40, 85100 Potenza, Italy; 4 Department of Life and Environmental Sciences, University of Cagliari, S.P. 8 Km 0.700, 09042 Monserrato, Italy; caddeoc@unica.it; 5 Spinoff TNcKILLERS s.r.l., Via dell’Ateneo Lucano 10, 85100 Potenza, Italy

**Keywords:** Box-Behnken design, black tea extract, transfersomes, photoaging prevention, dermal fibroblasts, LC-MS/MS

## Abstract

**Background/Objectives:** Ultraviolet B (UVB) radiation contributes significantly to skin aging and skin disorders by promoting oxidative stress, inflammation, and collagen degradation. Natural antioxidants such as theaflavins and thearubigins from *Camellia sinensis* L. (black tea) have shown photoprotective effects. This study aimed to optimize the extraction of theaflavins and thearubigins from black tea leaves and evaluate the efficacy of the extract against UVB-induced damage using a transfersome-based topical formulation. **Methods:** Extraction of theaflavins and thearubigins was optimized via response surface methodology (Box-Behnken Design), yielding an extract rich in active polyphenols. This extract was incorporated into transfersomes that were characterized for size, polydispersity, zeta potential, storage stability, and entrapment efficiency. Human dermal fibroblasts (NHDF) were used to assess cytotoxicity, protection against UVB-induced viability loss, collagen degradation, and expression of inflammatory (IL6, COX2, iNOS) and matrix-degrading (MMP1) markers. Cellular uptake of the extract’s bioactive marker compounds was measured via LC-MS/MS. **Results:** The transfersomes (~60 nm) showed a good stability and a high entrapment efficiency (>85%). The transfersomes significantly protected NHDF cells from UVB-induced cytotoxicity, restored collagen production, and reduced gene expression of MMP1, IL6, COX2, and iNOS. Cellular uptake of key extract’s polyphenols was markedly enhanced by the nanoformulation compared to the free extract. **Conclusions:** Black tea extract transfersomes effectively prevented UVB-induced oxidative and inflammatory damage in skin fibroblasts. This delivery system enhanced bioavailability of the extract and cellular protection, supporting the use of the optimized extract in cosmeceutical formulations targeting photoaging and UV-induced skin disorders.

## 1. Introduction

The skin represents the outermost layer of the human body, offering protection from harmful substances and ultraviolet (UV) radiation (315–280 nm). Direct and continuous exposure to UVB rays may induce skin damage, thus impairing its barrier function and causing the outbreak of different conditions like erythema, inflammation, edema, and skin tumours [1]. Excessive exposure to UVB radiation can also cause a reduction in skin elasticity due to the overproduction of reactive oxygen species (ROS), which trigger a cascade of molecular events that promote inflammation, DNA damage, and the development of various skin disorders [2]. ROS are also responsible for the degradation of collagen and other structural proteins in the skin [3], thereby significantly contributing to premature skin aging. Ultraviolet organic filters are the most investigated photoprotection measure for ultraviolet radiation’s adverse effects. However, these organic compounds may cause long-term side effects on human health. Several investigations have demonstrated the existence of three different routes of exposure to organic UV filters (dermal, inhalation, and oral routes) that may be related to endocrine-disrupting properties [4]. For instance, elevated urinary concentrations of benzophenone-3 (BP-3), an organic UV filter, were associated with decreased levels of testosterone in male adolescents [5], reduced luteinizing hormone, follicle-stimulating hormone, and estradiol in healthy women [6], and decreased triiodothyronine levels in pregnant women [7]. In addition, a correlation was found between increased biomarkers of urinary oxidative stress, 8-isoprostane and 8-hydrodeoxyguanosine, and urinary BP-3 [8]. Considering this evidence, other strategies to protect the skin from UV radiation damage involve the addition of natural antioxidants (such as polyphenols, *β*-carotene, or lycalcone A) to sunscreen formulas or oral supplementation of antioxidants [3]. Thanks to their antioxidant activity, medicinal plants have traditionally been employed to treat skin problems since increased oxidative stress, and thus radical species, are known to be the main cause of skin-threatening changes [3].

Among the wide range of natural products, black tea (*Camellia sinensis* L.) is largely regarded as one of the three top non-alcoholic drinks. About 75% of global tea intake is black tea [9] that is produced through green tea leaf fermentation and contains substantially lower levels of flavan-3-ol monomers due to peroxidase- and oxidase-mediated polyphenol conversion to theaflavins and greater amounts of thearubigins [10]. Theaflavins are characterized as dimer-like structures belonging to benzophenone molecules, with several phenolic hydroxyl groups made by tea polyphenol oxidation, while thearubigins are highly complex blends of high molecular weight polymers. These compounds represent the bioactive molecules characteristic of black tea, whose intake has been correlated to health benefits [10]. Acting as antioxidants, theaflavins are noted as tea’s “soft gold,” capable of preventing cardiovascular disease and lowering blood lipids. The antioxidant mechanisms of theaflavins mainly involve direct free radical scavenging, activation of the body’s antioxidant system, complexation with metal ions, inhibition of low-density lipoprotein oxidation, and enhancement of the activity of antioxidant enzymes [11]. As stated before, during the UV radiation process, the skin free radicals chain reaction is stimulated. An in vitro study has demonstrated that theaflavins possess anti-UVB damage capabilities. In particular, it was observed that theaflavins were able to absorb UV radiation in human epidermal keratinocytes (HaCaT) leading to a downregulation of NF-κB inflammation pathways, inhibition of cytotoxic aggregates formation, and increase in mitochondrial membrane potential [11]. On the other hand, thearubigins were found to reduce cell proliferation and inhibit chemical carcinogenesis of skin in mice by decreasing signalling kinase activities [12].

Moreover, the incorporation of plant extracts into phospholipid-based nanocarriers can significantly enhance their skin penetration and bioavailability, representing an effective strategy for the topical delivery of natural antioxidants in cosmeceuticals [13]. Transfersomes have been proposed to overcome the skin barrier layer and promote active molecules penetration. Transfersomes, which are composed of phospholipids and an edge activator with membrane-softening properties (such as Tween 80, Span 80, and sodium cholate), can accommodate a variety of natural compounds, regardless of their size, structure, or solubility [14]. Moreover, being highly deformable vesicles, transfersomes can squeeze through the narrow pores of the skin’s stratum corneum, allowing for deeper penetration and improved efficacy of carried biologically active compounds [15].

Considering this background, the present investigation aims to optimize the extraction of theaflavins and thearubigins from black tea by applying the response surface methodology built according to the Box-Behnken design, which was, to the best of our knowledge, carried out for the first time. This resulted in an optimized black tea extract that was incorporated into transfersomes tailored for skin application. The efficacy of black tea against UVB-induced collagen degradation, inflammation and extracellular matrix degradation was investigated in skin fibroblasts.

## 2. Materials and Methods

### 2.1. Materials

Caffeine (CAS No. 58-08-2), epigallocatechin gallate (EGCG, CAS No. 989-51-5), ethyl acetate (CAS No. 141-78-6), methanol (CAS No. 67-56-1), dimethyl sulfoxide (DMSO, CAS No. 67-68-5), isopropanol (CAS No. 67-63-0), formic acid (CAS No. 64-18-6), acetonitrile (CAS No. 75-05-8), sodium bicarbonate (NaHCO_3_, CAS No. 144-55-8), sodium hydroxide (NaOH, CAS No. 1310-73-2), hydrochloric acid (HCl, CAS No. 7647-01-0), and oxalic acid (CAS No. 144-62-7) were purchased from Sigma-Aldrich (Milan, Italy), unless otherwise stated. Lecithin from non-GMO soybean seeds, a mixture of phospholipids with 20–27% phosphatidylcholine, 16–22% phosphatidylethanolamine, 12–18% phosphatidylinositol, 3–9% phosphatidic acid, 3–4% phosphatidylserine, ≤4% lysophosphatidylcholine (CAS number: 8002435), and Tween 80 (polysorbate 80, HLB 15, CAS number: 9005-65-6) were purchased from Galeno (Carmignano, Italy). Commercial dried black tea (*Camellia sinensis* L.) leaves were purchased in a local market.

### 2.2. Extraction Optimization by Response Surface Methodology

The optimization of extraction conditions of theaflavins and thearubigins from black tea leaves was performed by applying the response surface methodology built according to the Box-Behnken design which comprised 3^3^ factorial levels (3 levels and 3 factors). The factor studies were selected based on previous investigations [16,17]. Three factors (temperature, drug concentration, and solvent) and three responses (extraction yield, theaflavins and thearubigins percentages) were chosen. The design was built using DesignExpert13 (Design-Expert Software v. 13, Stat-Ease Inc., Minneapolis, MN, USA) and consisted of 17 runs comprising 5 centre points. Table 1 reports the coded and the actual levels used in the design. Extraction time was set at 105 min based on preliminary experiments.

Each extraction was performed in triplicate using a water bath. Following the extraction process, the solutions underwent filtration, and the solvent was eliminated using a rotary evaporator set at 37 °C. The resulting dried extracts were stored in the dark at room temperature until use. The yields of the extraction were calculated as percentages using the formula shown below:

(1) $$\%=\frac{dried\;extract\;(g)}{dried\;black\;tea\;leaves\;(g)}\cdot 100$$

The effects of independent variables on the dependent ones were analysed using a second-order polynomial equation, and a multiple regression analysis was performed to establish a model linked to the factors being studied. The second-order polynomial equation was the following:

(2) $$Y\;=\;\beta_{0}\;+\;\sum_{i=1}^{3}\beta_{i}X_{i}+\sum_{i=1}^{3}\beta_{ii}X_{i}^{2}+\sum_{i=1}^{2}\sum_{j=i+1}^{3}\beta_{ij}X_{i}X_{j}$$

where Y is the dependent variable, *β*_0_ is the regression coefficient for the intercept, *β_i_*, *β_ii_*, and *β_ij_* are the regression coefficients for the linear, quadratic, and interaction influence of the independent variables *X_i_* and *X_j_* [18].

The goodness of the second-order polynomial model was evaluated by considering results from the ANOVA test, including the Lack of Fit, coefficient of determination (R^2^), adjusted coefficient of determination (Adj. R^2^), predicted coefficient of determination (Pred. R^2^), coefficient of variance (CV%), and adequate precision. Each polynomial equation was represented as a contour plot and surface plot to visualize the relationship between the experimental levels and the responses.

### 2.3. Process Optimization

Considering the results from the Box-Behnken design, the process optimization was carried out by applying the numerical optimization included in DesignExpert13. Once the optimized extraction conditions were discovered, they were validated experimentally to obtain an optimized black tea extract (BT).

### 2.4. Spectrophotometric Determination of Thearubigins and Theaflavins

For the spectrophotometric analysis of thearubigins and theaflavins [19], 50 mg of BT was dissolved in 50 mL of water and mixed with 50 mL of ethyl acetate. Then, 4 mL of the ethyl acetate layer was taken and brought to 25 mL with MeOH (Solution A). Another 25 mL of the ethyl acetate layer was taken and added to 25 mL of a 2.5% aqueous solution of NaHCO_3_. The aqueous layer was removed, and 4 mL of the washed ethyl acetate solution was brought to 25 mL with MeOH (Solution B). Then, 2 mL of a saturated aqueous solution of oxalic acid and 6 mL of water were added to 2 mL of the aqueous layer taken from the first extraction with ethyl acetate, and the solution was then brought to 25 mL with MeOH (Solution C). The absorbance of solutions A, B, and C was determined at 380 nm. For thearubigins and theaflavins determination, the following equations were used:

(3) $$\%\;\mathrm{Theaflavins}\;=\;6.25\;\cdot \;E_{c}\;\cdot \;f_{1}$$

(4) $$\%\;\mathrm{Thearubigins}=[(12.5\;\cdot \;E_{c}+6.25(E_{A}\;-\;E_{B})]\;\cdot \;f_{2}$$

where E_C_, E_A_, and E_B_ are the optical densities of Solutions A, B, and C at 380 nm, while f_1_ and f_2_ are spectrophotometric factors calculated as follows:

(5) $$f_{1}\;=\;\frac{0.02\;\cdot \;856.7\;\cdot \;extraction\;volume}{2.225\;\cdot \;892.7\;\cdot g\;of\;black\;tea\;extract}$$

(6) $$f_{2}\;=\frac{0.02\;\cdot \;extraction\;volume}{0.733\;\cdot g\;of\;black\;tea\;extract}$$

### 2.5. Transfersomes’ Production

The transfersomes were prepared according to a previously describe procedure [20,21] by dispersing lecithin in ultrapure water with Tween 80, in the presence or absence of BT, according to the amounts reported in Table 2, then sonicating this dispersion for 8 alternating cycles for 5 sec using an ultrasonic disintegrator (Soniprep 150 plus; MSE Crowley, London, UK).

### 2.6. Transfersomes’ Size, Homogeneity, Charge, Entrapment Efficiency and Storage Stability

The mean diameter, polydispersity index, and zeta potential of the transfersomes were measured via dynamic and electrophoretic light scattering (Zetasizer nano-ZS; Malvern Panalytical, Worcestershire, UK). For the measurements, samples were diluted (1:100 *v*/*v*) with ultrapure water and analysed at 25 ± 0.1 °C.

The black tea extract transfersomes (BTT, 1 mL; *n* = 4) were dialyzed against water (2 L) to remove the non-incorporated BT compounds. After 2 h under gentle stirring, both non-dialyzed and dialyzed transfersome dispersions were diluted (1:50 *v*/*v*) with 60:40 methanol:water to disrupt the vesicles and allow quantification via LC-MS/MS (see Section 2.14) of extract’s marker compounds, i.e., caffeine and epigallocatechin gallate. The entrapment efficiency of the BT transfersomes was calculated as the percentage of caffeine and epigallocatechin gallate in dialyzed *vs*. non-dialyzed transfersomes.

The stability of the BT transfersomes was studied by measuring their mean diameter, polydispersity index, and zeta potential during 5 months of storage at 4 ± 1 °C.

### 2.7. Transfersomes’ Ultrastructure

The ultrastructure of the BT transfersomes was investigated via cryogenic-Transmission Electron Microscopy (cryo-TEM). The vesicle dispersions (4 μL) were deposited onto glow-discharged EM grids, blotted, and vitrified by freezing into ethane using an automatic plunge freezer (Vitrobot MarkIV, FEI Company/ThermoFisher Scientific, Waltham, MA, USA). The vitrified samples were observed on a 200 kV electron microscope (Glacios2, FEI). Micrographs were taken at a magnification of 92,000× under low electron dose conditions on a Falcon 4i direct electron detector (ThermoFisher Scientific).

### 2.8. Cell Culture

The Normal Human Dermal Fibroblast cell line (NHDF; Cat. no. C-12302) and fibroblast growth medium 2 (Cat. no. C-23020) were purchased from PromoCell GmbH (Heidelberg, Germany).

Cells were grown in their culture medium supplemented with foetal calf serum (2% *v*/*v*), basic fibroblast growth factor (1 ng/mL), human recombinant insulin (5 μg/mL), penicillin (100 U/mL), and streptomycin (0.1 mg/mL) at 37 °C in a humidified incubator containing 5% CO_2_.

### 2.9. UVB Irradiation

NHDF cells were pre-treated with different concentrations (1–200 μg/mL) of BT in DMSO solution and BT in transfersomes for 24 h. The medium was removed, and the cells were washed with Phosphate-buffered saline (PBS; P2272), then irradiated under a thin layer of PBS with UVB (100 mJ/cm^2^) using a Bio-Link BLX-312 (Vilber Lourmat GmbH, Marne-la-Vallée, France). After irradiation, serum-free medium was added for additional 24 h. Non-irradiated cells were used as a control [22].

### 2.10. Cell Viability Assay

The colorimetric 3-(4,5-dimethyl-2-thiazolyl)-2,5-diphenyl-2*H*-tetrazoliumbromide (MTT; CAS number: 298-93-1) assay was used to determine the cell viability. NHDF cells were seeded in a 96-well plate (1 × 10^4^ cells/well), incubated overnight, and treated with different concentrations (1–200 μg/mL) of BT in DMSO solution, BT transfersomes, and ET for 24 h, followed or not by UVB exposure. MTT solution (0.75 mg/mL) was added to the wells and incubated for 4 h. Formazan crystals produced by viable cells were dissolved using DMSO:isopropanol (1:1). Absorbance was measured at 560 nm using a UV–Vis spectrophotometer (SPECTROstar^Nano^ BMG Labtech, Ortenberg, Germany) [23].

### 2.11. Total Collagen Production

Collagen production was quantified by seeding NHDF cells into 96-well plates (1 × 10^4^ cells/well) and pre-treating them with BT in DMSO solution and BT in transfersomes (1–200 μg/mL). After 24 h of pre-treatment, the cells were exposed to UVB irradiation, as described above. After incubation of cells in serum-free medium, 4% formaldehyde in PBS was used to fix the cells overnight. Collagen was stained for 60 min with 50 µL/well Sirius Red solution (Cat. no. ab246832; Abcam, Cambridge, UK). After the incubation period, the dye solution was aspirated and the cells were washed with 100 μL of 0.1 M HCl, then the dye was eluted in 100 μL of 0.1 M NaOH. Absorbance was measured at 510 nm using a UV–Vis spectrophotometer (SPECTROstar^Nano^ BMG Labtech, Ortenberg, Germany) [24].

### 2.12. Quantitative Real-Time PCR

NHDF cells were seeded into 6-well plates (3 × 10^5^ cells/well) and were pre-treated with BT in DMSO solution and BT in transfersomes (10 and 100 μg/mL) for 24 h, and then cells underwent UVB irradiation as described above. Total RNA was extracted from treated or untreated cells using the RNeasy Mini Kit (Cat.no 74106; QIAGEN, Hilden, Germany) and transcribed to cDNA using the high-capacity cDNA reverse transcription kit (Thermo Fisher Scientific, Miami, FL, USA), according to the manufacturer’s instructions. The strands obtained were used as templates for quantitative real-time PCR (qRT-PCR) using SYBR Green qPCR master mix (Cat. no. 4309155; Thermo Fisher Scientific, Monza, Italy) and a 7500 Fast Real-Time PCR System (Applied Biosystems, Foster City, CA, USA) [25]. *β*-Actin mRNA levels were used as internal controls. The primers used in this study were the following: *β*-actin, forward 5′-CCTGGCACCCAGCACAAT-3′ and reverse 5′-GCCGATCCACACGGAGTACT-3′; MMP-1, forward 5′-CTGGCCACAACTGCCAAAT-3′ and reverse 5′-CTGTCCCTGAACAGCCCAGTACTTA-3′; IL-6, forward 5′-CTACTCTCAAATCTGTTCTGG-3′ and reverse 5′-GGATTCAATGAGGAGACTTG-3′; COX-2, forward 5′-CAGCACTTCACGCATCAGTT-3′ and reverse 5′-CAGCAAACCGTAGATGCTCA-3′; iNOS, forward 5′-AGAGAGATCGGGTTCACA-3′ and reverse 5′-CACAGAACTGAGGGTACA-3′.

### 2.13. Cellular Uptake of Black Tea Extract

To evaluate the cellular uptake of black tea extract, NHDF cells were seeded into 6-well plates (3 × 10^5^ cells/well) and treated with BT in DMSO solution or in transfersomes (10 and 50 ug/mL). After 24 h, the cells were trypsinized, pelleted at 1200 rpm for 5 min, and the supernatant was removed. To obtain the lysates, 500 µL of 40% MeOH with 0.1% *v*/*v* formic acid was added to the cell pellets. The pellets were then sonicated (1 min on/1 min off for 10 min), mixed, and left on ice for 15 min. Lysates were centrifuged at 1500 rpm for 5 min, and the supernatants were collected for LC-MS/MS analysis [20].

### 2.14. LC-MS/MS Analysis

To determine the cellular uptake of the extract, two key markers (caffeine and epigallocatechin gallate) were quantified. The supernatants collected from the cell lysates were dried and dissolved in 100 µL of water, and a 10 µL aliquot was injected for HR-ESI-LC-MS/MS analysis. Analyses were carried out on an ABSCIEX API 6500 QTRAP^®^ mass spectrometer (Foster City, CA, USA), operating in both positive and negative ion modes, coupled to a Nexera X2 UPLC Shimadzu system (Shimadzu, Milan, Italy). Chromatographic separation was performed on a Luna^®^ Omega column (100 × 1.6 mm, 3 µm, 100 Å; Phenomenex^®^, Castel Maggiore, Bologna, Italy) using a mobile phase consisting of water with 0.1% formic acid (solvent A) and acetonitrile (solvent B). The gradient consisted of an initial isocratic phase at 2% B for 2.5 min, followed by a rapid increase to 70% B over 10 min, ending with a final wash. The flow rate was set at 0.25 mL/min and the column temperature was maintained at 30 °C. A multiple reaction monitoring method was used to monitor the transitions *m*/*z* 457→169 for epigallocatechin gallate and *m*/*z* 195→138 for caffeine [21].

### 2.15. Statistical Analysis

Data are expressed as means ± standard deviations (SD). GraphPad Prism 8 Software, Inc. (San Diego, CA, USA) was used for the statistical analysis, and *p* values < 0.05 were considered as statistically significant.

## 3. Results

### 3.1. Response Surface Methodology Model Adequacy

Applying the response surface methodology, the Box-Behnken design matrix was built (Table 3) to investigate the effect of temperature 50–65–80 °C (A), concentrations 0.0250–0.0625–0.1000 g/mL (B), and solvent 0–40–80 %EtOH/H_2_O (C) on black tea extraction yield, and the percentage of theaflavins (%TF) and thearubigins (%TR) was determined spectrophotometrically.

The ANOVA test was applied to determine the influence of the analysed variables on the response, their interactions, and the statistical significance of the model (Table 4); in all cases, the data were not transformed based on the Base 10 Log, Natural Log, or Square Roots. The statistical significance of the quadratic model was evaluated considering the F-value and the *p*-value. As shown in Table 4, the high F-value and low *p*-value for all dependent variables indicate the quadratic model adequacy. This was also confirmed by the Lack of Fit since, in all cases, it was not significant, indicating that the applied model fits the data well.

The reliability of the quadratic model fit was evaluated considering the coefficient of determination (R^2^), adjusted coefficient of determination (Adj. R^2^), predicted coefficient of determination (Pred. R^2^), coefficient of variance (%CV), and adequate precision (Table 5). For all dependent variables considered, high R^2^ values were obtained (0.9891, 0.9628, 0.9798 for extraction yield, %TF, and %TR, respectively) and a difference lower than 0.2 between the Adj. R^2^ and the Pred. R^2^, thus confirming the model adequacy. Furthermore, an Adequate Precision greater than four indicates that the statistical model can distinguish signal (i.e., the effects of significant factors) from experimental noise (random variability) well, thus suggesting that the model has a good predictive ability, which is also useful outside the range of the original data (Table 5).

**Figure 1:**
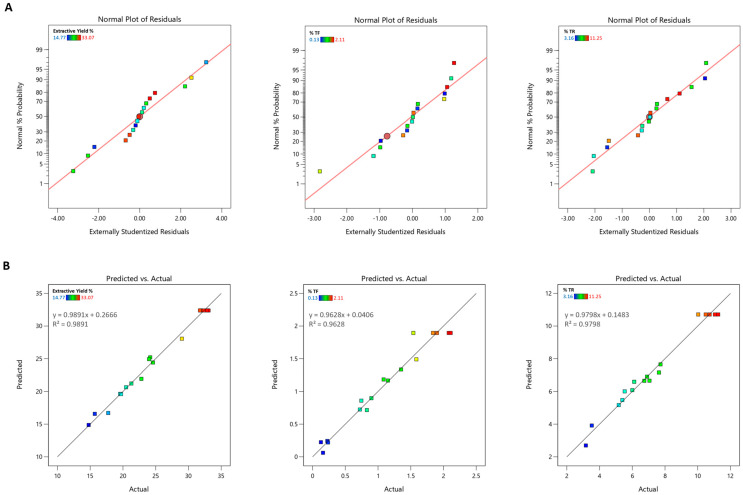
(**A**) Normal plot of residuals for extraction yield, percent of theaflavins (%TF), and percentage of thearubigins (%TR). (**B**) Predicted *vs*. actual plots for extraction yield, percentage of theaflavins (%TF), and percentage of thearubigins (%TR).

Finally, the normal probability plots of the residuals (Figure 1A) show a nearly linear distribution, confirming that the residuals follow a normal distribution, an essential requirement for the validity of the response surface methodology model. On the other hand, the “Predicted *vs*. Actual” plots (Figure 1B) show the excellent alignment of the points along the bisector. These results indicate that the developed model is highly predictive and reliable in describing the relationship between the independent variables and the measured responses.

### 3.2. Effect of Extraction Parameters on Dependent Variables

The second-order polynomial equations for each response were used to identify the independent variables’ effect on black tea extraction yield, TF, and TR percentages (Table 6).

**Figure 2:**
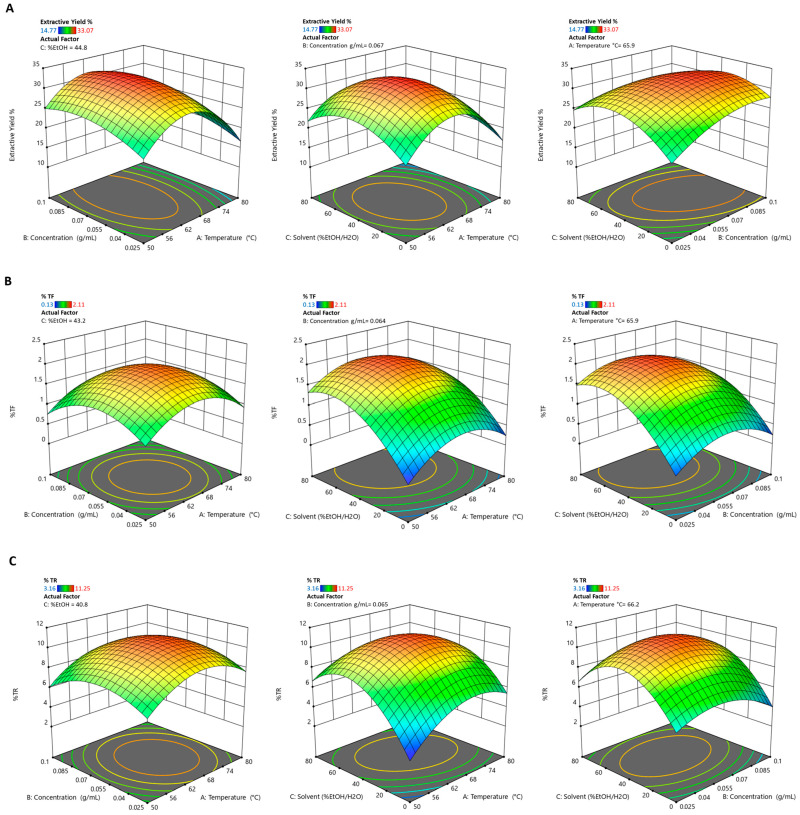
Surface plot and contour plot showing the effects of temperature, concentration, and solvent on (**A**) extraction yield, (**B**) % of theaflavins (%TF), and (**C**) % of thearubigins (%TR).

Regarding the effect that the selected extraction variables have on the extraction yield (Table 6 and Figure 2A), it was seen that temperature (A) had a highly significant negative effect (coeff. = −2.52, *p*-value = 0.0002), which indicates that an increase in temperature tends to reduce the extraction yield. In contrast, concentration (B) showed a significant positive effect (coeff. = +1.72, *p*-value = 0.0017), which suggests that higher concentrations promote yield. The % EtOH/H_2_O (C) had no significant impact (*p*-value = 0.6665). On the other hand, the % of theaflavins is strongly influenced by % EtOH/H_2_O (C), with a significant positive coefficient (coeff. = +0.553, *p*-value < 0.0001), which indicates that an increase in the ethanol component favours theaflavins extraction. Variables A (temperature) and B (concentration) showed no significant effects in the linear terms (Table 6 and Figure 2B). Likewise, for thearubigins, the most significant effect is % EtOH/H_2_O (C), with a significant positive coefficient (coeff. = +1.12, *p*-value = 0.0007). Temperature (A) also has a positive effect (*p*-value = 0.0293), while concentration (B) is not significant. Among the interactions, the A-C term (Temperature × EtOH/H_2_O) is significantly negative (*p*-value = 0.0173), which indicates an antagonistic interaction between these two variables (Table 6 and Figure 2C). In all responses, the quadratic terms A^2^, B^2^, and C^2^ were found to be significant (*p*-value < 0.05), which indicates the parabolic behaviour of the response. This suggests the existence of an optimal point within the experimental domain, rather than at the extremes.

### 3.3. Multiple Response Optimization

Considering the data obtained, the optimal extraction conditions necessary to obtain extracts with the highest extraction yield and the highest content of theaflavins and thearubigins were determined to be 64.214 °C, 0.063 g/mL, 48.745% EtOH/H_2_O (Desirability 0.945). Table 7 shows the predicted values for each response that would be obtained by applying the optimal extraction conditions obtained from the applied model.

The predicted optimal extraction conditions were used to obtain the optimized extract, which resulted in an extraction yield of 33.029 ± 1.603%, 10.961 ± 0.0289 %TF, and 2.365 ± 0.1021 %TR. It is evident that all the dependent variables evaluated fall within the predicted range, further confirming the validity of the constructed model.

### 3.4. Transfersomes’ Production and Characterization

A transfersome-based formulation was developed for the skin delivery of BT. The transfersomes were characterized for mean diameter, homogeneity, and surface charge and compared with empty transfersomes (Table 8). The BT transfersomes were around 60 nm, homogeneous in size (0.25 of polydispersity index), and negatively charged (−40 mV). The loading of the extract affected only the polydispersity index, which was lower for the empty transfersomes (0.23, *p* < 0.001; Table 8). Nevertheless, both values indicate a uniform nanoparticle size, as opposite to values greater than 0.3, which suggests some degree of polydispersity.

The entrapment efficiency of the BT transfersomes, which was calculated based on the amounts of caffeine and epigallocatechin gallate that are main components of the extract, was high: 86 ± 2.3% and 93 ± 4.8%, respectively.

The storage stability of BT transfersomes was estimated via periodic measurements of their mean diameter, polydispersity index and zeta potential. During five months, the transfersomes showed minimal variations in size and homogeneity (Table 9), indicating high stability of the nanoformulation.

**Figure 3:**
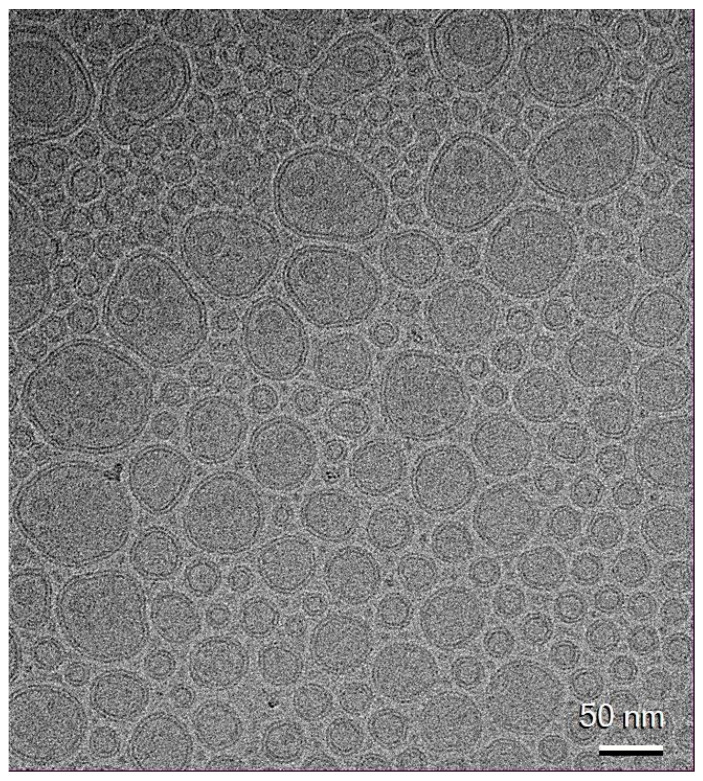
Cryo-TEM micrograph of black tea transfersomes at 92,000× magnification.

Cryo-TEM micrographs of BT transfersomes (Figure 3) displayed fairly spherical, unilamellar structures well below 100 nm in size, in alignment with dynamic light scattering data (Table 8).

### 3.5. Cell Viability Assessment

**Figure 4:**
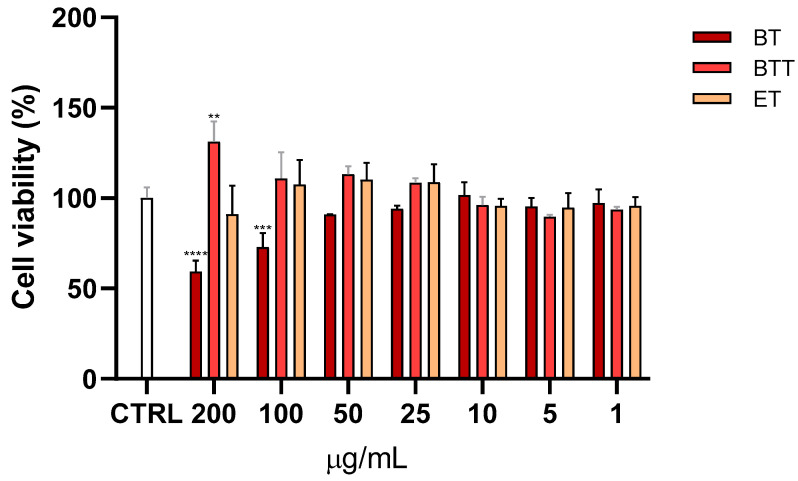
Cell viability, evaluated by MTT assay, of Normal Human Dermal Fibroblasts (NHDF) treated for 24 h with different concentrations (1–200 μg/mL) of black tea extract in solution (BT) or in transfersomes (BTT) and empty transfersomes (ET). Data are expressed as the means ± SD of three independent experiments (*n* = 3) and were analysed by one-way ANOVA followed by Tukey’s post-hoc test. ** *p* < 0.01, *** *p* < 0.001, **** *p* < 0.0001, *vs*. CTRL (100% of viability).

The MTT test was utilized to evaluate cell viability because it quantifies how the cellular mitochondrial dehydrogenase enzyme, which is only active in live cells, converts the yellow, water-soluble substrate MTT into purple formazan crystals. The effects of different concentrations (1–200 μg/mL) of BT in solution or in transfersomes, or empty transfersomes on the viability of NHDF cells was evaluated. The cell viability was measured after 24 h of treatment and is presented as a percentage relative to the control group (CTRL), which represents 100% viability (Figure 4). At higher concentrations (200 and 100 μg/mL), BT significantly reduced cell viability compared to the control, while the cytotoxicity was prevented by the incorporation into the transfersomes. Empty transfersomes maintained cell viability close to the control levels across all tested concentrations, indicating that the nanocarrier itself did not affect cell viability.

**Figure 5:**
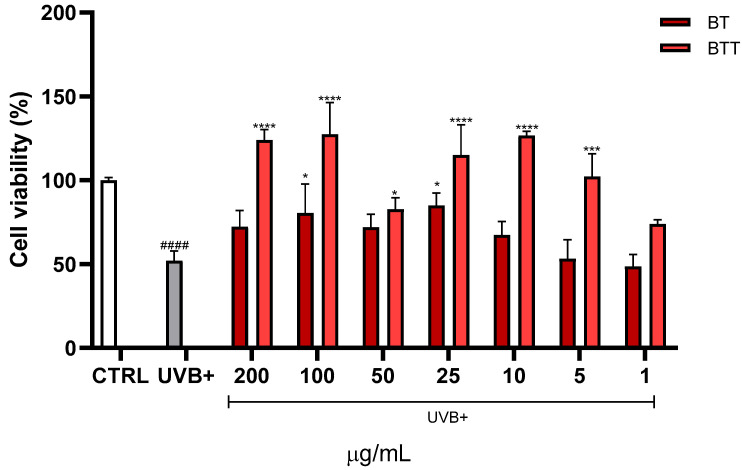
Cell viability, evaluated by MTT assay of Normal Human Dermal Fibroblasts (NHDF) treated for 24 h with different concentrations (1–200 μg/mL) of black tea extract in solution (BT) or in transfersomes (BTT), then irradiated with UVB (100 mJ/cm^2^). Data are expressed as the means ± SD of three independent experiments (*n* = 3) and were analysed by one-way ANOVA followed by Tukey’s post-hoc test. **** *p* < 0.0001, *** *p* < 0.001, * *p* < 0.05 *vs*. UVB+, ^####^ *p* < 0.0001 *vs*. CTRL (100% of viability).

The protective effects of BT and BT transfersomes against UVB-induced loss of viability was investigated. MTT assay was performed after treating NHDF cells with increasing concentrations of the extract (1–200 μg/mL) before UVB exposure (Figure 5). UVB exposure significantly decreased cell viability compared to the CTRL group (*p* < 0.0001). Notably, BT transfersomes provided a significantly greater protection at 100 μg/mL and 200 μg/mL compared to BT in its free form (i.e., in DMSO solution).

### 3.6. Collagen Degradation

**Figure 6:**
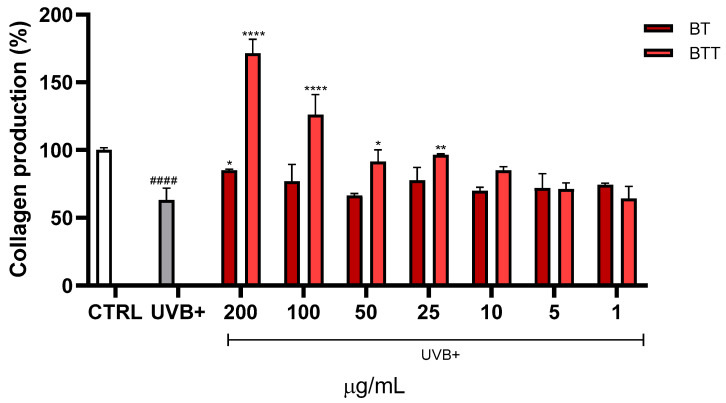
Collagen production was evaluated using Sirius red staining of Normal Human Dermal Fibroblasts (NHDF) treated for 24 h with different concentrations (1–200 μg/mL) of black tea extract in solution (BT) or in transfersomes (BTT), then irradiated with UVB (100 mJ/cm^2^). Data are expressed as the mean ± SD of three independent experiments (*n* = 3) and were analysed using one-way ANOVA, followed by Tukey’s post-hoc test. **** *p* < 0.0001, ** *p* < 0.01, * *p* < 0.05 *vs*. UVB+, ^####^ *p* < 0.0001 *vs*. CTRL (100% collagen production).

Collagen fibres were stained using Sirius red. UVB exposure led to a marked reduction in collagen production, with levels dropping to approximately one half compared to the untreated cells (CTRL) (Figure 6). Treatment with BT partially restored collagen levels. Treatment with BT transfersomes demonstrated superior efficacy compared to free BT, not only protecting from UVB damage but even enhancing collagen synthesis at 100 μg/mL and 200 μg/mL.

### 3.7. Gene Expression Analysis

**Figure 7:**
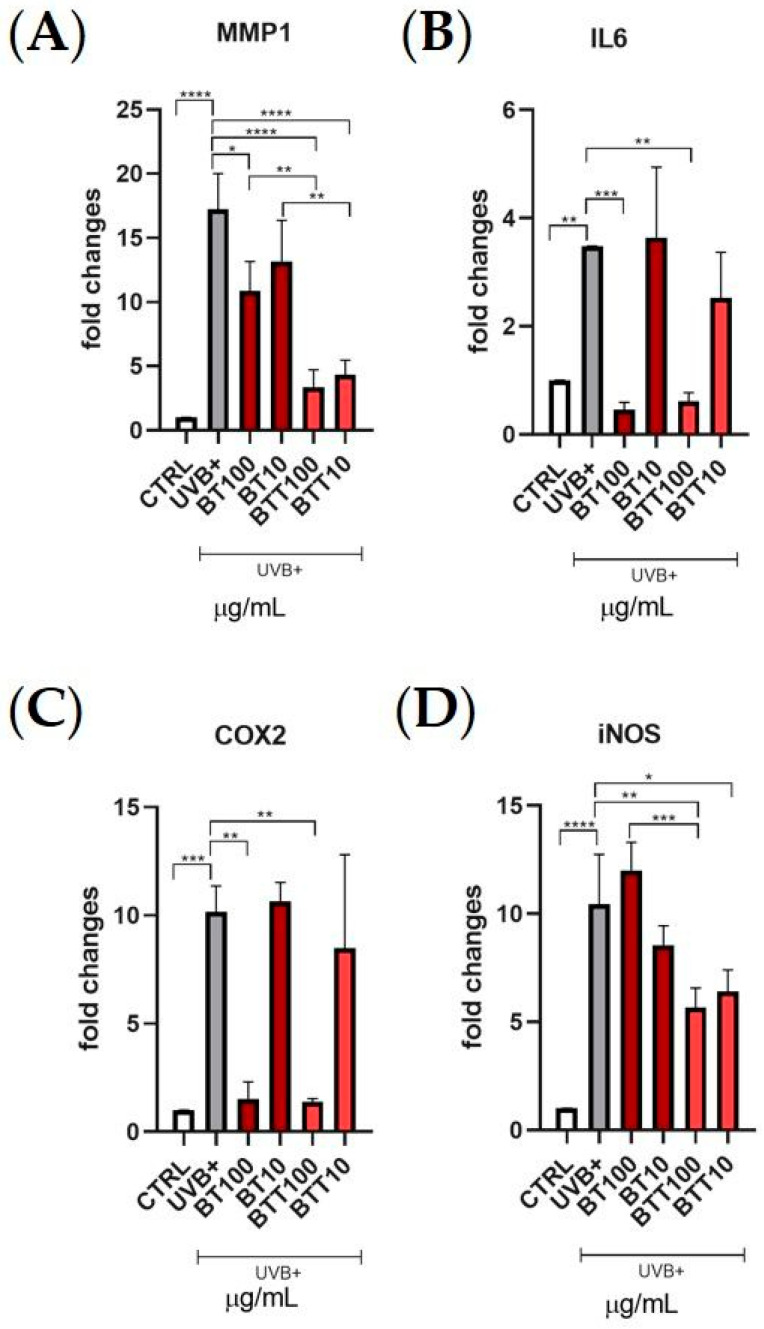
Normal Human Dermal Fibroblasts (NHDF) were treated for 24 h two concentrations (10 and 100 μg/mL) of black tea extract in solution (BT) or in transfersomes (BTT), then irradiated with UVB (100 mJ/cm^2^). Gene expression of MMP1 (**A**), IL6 (**B**), COX2 (**C**), and iNOS (**D**) was analysed by real time PCR normalized with the housekeeping gene *β*-actin. Data are expressed as the means ± SD of three independent experiments (*n* = 3) and were analysed by one-way ANOVA followed by Tukey’s post-hoc test. **** *p* < 0.0001, *** *p* < 0.001, ** *p* < 0.01, * *p* < 0.05.

Collagen degradation is a hallmark of UVB-induced skin aging, driven primarily by increased expression of matrix metalloproteases and pro-inflammatory mediators. This study examined the expression levels of inflammatory and matrix-degrading markers MMP1, IL6, COX2, and iNOS in Normal Human Dermal Fibroblasts (NHDF) treated with BT solution or BT transfersomes at 10 µg/mL and 100 µg/mL, followed by UVB irradiation (Figure 7). The results showed that UVB exposure led to a significant increase in MMP1 expression compared to control levels. Treatment with BT in solution at 100 µg/mL significantly reduced MMP1 expression compared to the UVB+ group. The extract in the transfersomes showed a more pronounced reduction in MMP1 expression, lowering it to near control levels, at both concentrations. The UVB irradiation tripled the expression of IL6 in comparison with untreated control cells. Both BT solution and BT transfersomes treatments significantly suppressed IL6 expression at the higher concentration (100 µg/mL). Similarly, UVB exposure led to a 10-fold increase in COX2 expression and both BT solution and BT transfersomes at 100 µg/mL restored basal values. Lastly, UVB exposure significantly upregulated iNOS expression. Treatment with BT solution was basically ineffective, while BT transfersomes significantly reduced iNOS expression already at 10 µg/mL.

### 3.8. Intracellular Uptake of Black Tea Extract

**Figure 8:**
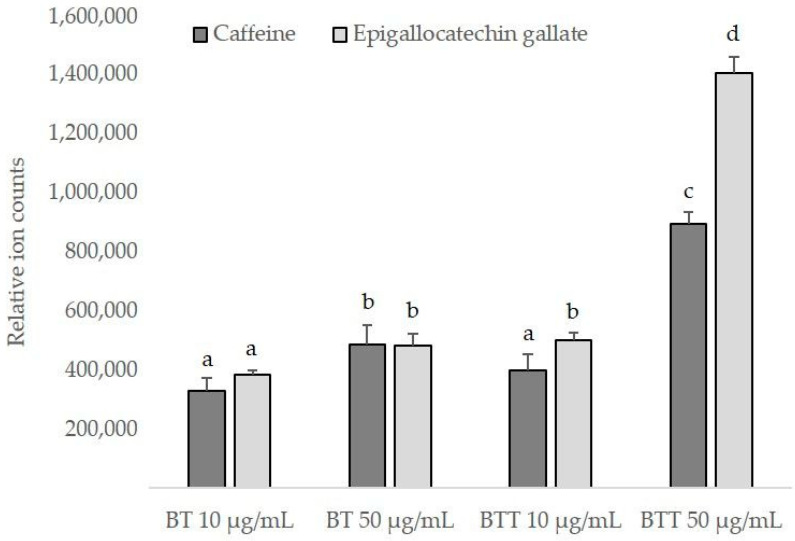
Amounts of caffeine and epigallocatechin gallate detected in NHDF cells after exposure to black tea extract in solution (BT) or in transfersomes (BTT). Two concentrations of the extract were tested (10 and 50 μg/mL). Results are the means ± standard deviations of three replicates; different letters (a–d) indicate statistically significant differences (*p* < 0.05).

The intracellular uptake of BT delivered by the transfersomes was assessed in comparison with that obtained after exposure of NHDF cells to the free extract. LC-MS/MS analyses were performed, and caffeine and epigallocatechin gallate were used as marker compounds for relative quantification purposes; in fact, these compounds are the main active ingredients in tea and are known for their physiological functions [26,27]. The results in Figure 8 show that there was a slight yet significant increased content of both markers using the free extract at the higher concentration (50 µg/mL). Similar values were found in the cells exposed to the lower concentration of BT in transfersomes (10 µg/mL). This means that BT transfersomes were basically as effective as BT on its own at a 5-times higher concentration. Furthermore, when BT was delivered by the transfersomes, at 50 µg/mL, the intracellular amounts of caffeine and epigallocatechin gallate were ~1.8- and ~3-fold higher than those found after BT solution application, at the same concentration.

## 4. Discussion

Excessive sun exposure is a leading cause of skin diseases. In particular, UVB radiation (280–315 nm) encourage ROS production [28], which damages DNA. Skin inflammation, photoaging, and photocarcinogenesis are the final results of this damage [29,30]. Polyphenolic compounds, thanks to their antioxidant capacity, have potential as therapeutic agents for a wide range of disorders, including skin conditions. In particular, two characteristic specialized molecules from black tea (*C. sinensis*), theaflavin and thearubigins, demonstrated the ability to inhibit photoaging and skin cells’ intrinsic aging [11,12].

For this reason, the present investigation aimed to optimize the extraction of theaflavins and thearubigins to obtain an optimized black tea extract. The response surface methodology showed that the % of theaflavins and thearubigins is strongly influenced by % EtOH/H_2_O with a significant positive coefficient, indicating that an increase in the ethanol component favours theaflavins extraction. However, the parabolic behaviour of the response suggests the existence of the optimal point within the experimental domain, predicted as optimal extraction condition of 64.214 °C, 0.063 g/mL, 48.745% EtOH/H_2_O. Compared to Wang *et al*. [28], who employed a conventional hot water infusion and reported a TF content of 0.24% and TR content of 4.07%, our extraction method resulted in a significantly higher TF yield (10.961 ± 0.0289%) and a slightly lower TR level (2.365 ± 0.1021%). These findings support the model’s prediction that ethanol selectively enhances the recovery of theaflavins, likely due to differences in solvent polarity and extraction efficiency.

This study provides substantial evidence that the optimized BT exerts protective effects against UVB-induced damage in skin fibroblasts by preventing the loss of viability, collagen degradation, and downregulating key mediators involved in inflammation and extracellular matrix degradation. These findings underscore the potential of BT as a natural agent for preventing UVB-induced skin damage (e.g., reduced cell viability and collagen production [31]), which is critical in the context of skin aging and the development of skin diseases, including cancer. Our outcomes are comparable with those of Xu et al. (2025) [1], who showed that polyphenolic compounds from black tea had photoprotective properties when administered orally to UVB-exposed mice. Despite using distinct models, both studies demonstrate reduced inflammation, and collagen preservation. These findings support the potential of tea-derived polyphenols to prevent UVB-induced skin damage. Notably, in our study, the treatment with BT transfersomes prevented or reduced all the investigated indicators of UVB-induced damage (i.e., cell mortality, collagen degradation, and increased expression of MMP1, IL6, COX2, and iNOS), providing significantly greater protection than the extract on its own. The increased efficacy may reasonably be due to the ability of the nanocarrier to facilitate the passage of the bioactive molecules across cell membranes [32], as demonstrated in this study by the higher intracellular amounts of BT marker compounds found in the cells treated with BT transfersomes than with BT solution. Our findings are consistent with other studies in which black tea extract was successfully incorporated into niosomal formulations for topical delivery and photoprotection [33], further supporting the potential of nanocarriers to enhance the absorption and protective effects of tea-derived compounds. Polyphenols in black tea, such as catechins and theaflavins, are known to enhance collagen synthesis and inhibit collagenase activity [34,35], which may explain the observed protection of collagen degradation following treatment with the extract. UVB radiation is also known to trigger oxidative stress and inflammatory response in the skin, leading to the upregulation of enzymes such as iNOS [36]. iNOS is responsible for producing high levels of nitric oxide in response to stress, which can contribute to oxidative damage and inflammation. The reduction in iNOS expression observed after the treatment with BT transfersomes is likely due to the high content of polyphenolic compounds in BT, which have been well documented for their antioxidant properties [37]. Matrix metalloproteinase-1 (MMP-1) is another critical enzyme that is upregulated by UVB exposure and is involved in the breakdown of collagen, leading to the degradation of the skin’s extracellular matrix and contributing to photoaging [38]. The significant reduction in MMP-1 expression in skin fibroblasts treated with BT indicates that this may help preserve the structural integrity of the skin by preventing collagen degradation. This aligns with recent research showing that polyphenolic compounds from various natural sources can inhibit UV-induced MMP-1 expression, thereby protecting against photoaging [34]. Cyclooxygenase-2 (COX-2) is a key enzyme in the inflammatory pathway, and its upregulation is closely associated with UVB-induced inflammation and carcinogenesis in the skin [39]. The downregulation of COX-2 and interleukin-6 (IL-6) suggests that BT may exert anti-inflammatory effects, reducing the risk of chronic inflammation and subsequent skin damage or cancer development [40,41]. These findings suggest that BT could be a valuable natural resource for preventing skin aging and reducing the risk of UV-induced skin disorders. Moreover, the formulation in transfersomes can expand BT’s applicability to cosmeceuticals [42]. Transfersomes can not only improve the solubility and stability of entrapped BT’s biologically active compounds, which leads to improved bioavailability, but also enhance their skin penetration. The improved bioavailability of transfersomal BT was demonstrated in this study by a higher intracellular accumulation and a greater effectiveness in preventing or reducing UVB-induced damage. Ultimately, the deformability and the small size (~60 nm) of the produced transfersomes are expected to promote BT’s skin penetration: smaller deformable vesicles generally penetrate the skin more efficiently due to their ability to traverse intercellular spaces reaching deeper layers. Future research should focus on in vivo studies and clinical trials to explore skin absorption and the cosmeceutical potential of BT in transfersomes.

## 5. Conclusions

This study demonstrates the successful optimization of black tea extract and its formulation in stable and efficient transfersomes for skin application. Using the Box-Behnken response surface approach, the extraction of black tea polyphenols, particularly theaflavins and thearubigins, was effectively optimized, with ideal conditions identified as 64.214 °C, 0.063 g/mL, 48.745% EtOH/H_2_O. Black tea bioactive compounds were efficiently loaded into transfersomes, which showed high entrapment efficiency and maintained physicochemical stability over five months. The transfersomes significantly boosted the extract’s efficacy against UVB-induced damage in human dermal fibroblasts. In particular, they preserved cell viability, restored collagen levels, and reduced key markers of inflammation (IL6, COX2, iNOS) and extracellular matrix degradation (MMP1). The nanocarrier system promoted the intracellular delivery of active compounds by 1.8- to 3-fold in cells treated with the nanoformulation compared to those treated with the free extract. These findings suggest that black tea extract transfersomes represent an effective phytotherapeutic approach for preventing photoaging and inflammatory skin damage. Further, in vivo and clinical investigations are warranted to validate these effects and explore their cosmeceutical potential.

## Figures and Tables

**Table 1:** Coded and actual levels used in the Box-Behnken design.

	Levels			
**Independent Variables**	**−1**	**0**	**1**	**Dependent Variables**
**A-Temperature °C**	50	65	80	Extraction yield (%)
**B-Concentration g/mL**	0.0250	0.0625	0.1000	Thearubigins (%)
**C-Solvent %EtOH/H_2_O**	0	40	80	Theaflavins (%)

**Table 2:** Composition of the transfersomes.

Formulation	Lecithin (mg)	Black Tea Extract (mg)	Tween 80 (mL)	H_2_O (mL)
**Empty transfersomes (ET)**	140	0	0.05	0.95
**Black tea transfersomes (BTT)**	140	5	0.05	0.95

**Table 3:** Box-Behnken design matrix showing the independent variable (A, B, C) and the dependent variables.

	Independent Variables			Dependent Variables		
	A	B	C	Extraction Yield %	%TF	%TR
Run	Temperature °C	Concentration g/mL	%EtOH/H_2_O			
1	65	0.0625	40	32.34 ± 1.026 ^a^	1.90 ± 0.010 ^b^	10.73 ± 0.260 ^a^
2	65	0.0250	80	23.99 ± 2.018 ^b^	1.59 ± 0.030 ^c^	7.05 ± 0.178 ^b,c^
3	50	0.1000	40	24.16 ± 1.791 ^b^	0.72 ± 0.010 ^g^	6.00 ± 0.044 ^c,d,e^
4	65	0.0625	40	31.93 ± 2.036 ^a^	2.08 ± 0.067 ^a^	10.03 ± 1.006 ^a^
5	80	0.0625	80	14.77 ± 0.031 ^f^	1.09 ± 0.007 ^e^	5.54 ± 0.087 ^d,e^
6	50	0.0250	40	21.29 ± 1.150 ^b,c,d^	0.75 ± 0.003 ^g^	6.12 ± 0.028 ^c,d,e^
7	65	0.0625	40	32.85 ± 0.691 ^a^	1.54 ± 0.004 ^c^	11.25 ± 1.093 ^a^
8	65	0.0250	0	20.48 ± 1.362 ^b,c,d^	0.24 ± 0.002 ^h^	5.17 ± 0.050 ^e^
9	65	0.0625	40	33.07 ± 2.107 ^a^	1.84 ± 0.081 ^b^	10.49 ± 0.099 ^a^
10	80	0.1000	40	19.57 ± 0.714 ^c,d,e^	0.83 ± 0.012 ^f^	7.63 ± 0.081 ^b^
11	50	0.0625	80	22.81 ± 1.740 ^b,c^	1.35 ± 0.004 ^d^	6.74 ± 0.034 ^b,c,d^
12	65	0.1000	80	24.58 ± 0.641 ^b^	1.15 ± 0.011 ^e^	6.90 ± 0.055 ^b,c^
13	65	0.1000	0	29.00 ± 0.532 ^a^	0.13 ± 0.001 ^i^	3.53 ± 0.058 ^f^
14	80	0.0250	40	17.75 ± 1.624 ^d,e,f^	0.90 ± 0.012 ^f^	7.73 ± 0.092 ^b^
15	80	0.0625	0	15.70 ± 0.763 ^e,f^	0.23 ± 0.000 ^h^	5.39 ± 0.046 ^e^
16	65	0.0625	40	31.76 ± 2.057 ^a^	2.11 ± 0.015 ^a^	11.05 ± 0.624 ^a^
17	50	0.0625	0	19.71 ± 0.681 ^c,d,e^	0.16 ± 0.007 ^h,i^	3.16 ± 0.038 ^f^

%TF: percentage of theaflavins; %TR: percentage of thearubigins; the results are reported as means ± standard deviations; different letters (a–i) indicate statistically significant differences (*p* < 0.05).

**Table 4:** ANOVA statistical analysis of the model.

Response 1: Extraction Yield %						
Source	Sum of Squares	df	Mean Square	F-Value	*p*-Value	
**Quadratic Model**	620.67	9	68.96	70.58	<0.0001	significant
A-Temperature	50.94	1	50.94	52.13	0.0002 *	
B-Concentration	23.8	1	23.8	24.36	0.0017 *	
C-Solvent	0.1977	1	0.1977	0.2023	0.6665	
AB	0.2797	1	0.2797	0.2863	0.6092	
AC	4.07	1	4.07	4.17	0.0806	
BC	15.72	1	15.72	16.09	0.0051	
A^2^	339.69	1	339.69	347.66	<0.0001 *	
B^2^	31.09	1	31.09	31.82	0.0008 *	
C^2^	112.13	1	112.13	114.76	<0.0001 *	
**Residual**	6.84	7	0.9771			
Lack of Fit	5.55	3	1.85	5.76	0.0619	not significant
Pure Error	1.29	4	0.3213			
**Corrected Total**	627.51	16				
**Response 2: %TF**						
**Source**	**Sum of Squares**	**df**	**Mean Square**	**F-Value**	***p*-Value**	
**Quadratic Model**	7.11	9	0.7902	20.15	0.0003	significant
A-Temperature	0.0005	1	0.0005	0.0117	0.917	
B-Concentration	0.0504	1	0.0504	1.29	0.2942	
C-Solvent	2.45	1	2.45	62.45	<0.0001 *	
AB	0.0005	1	0.0005	0.0139	0.9095	
AC	0.0275	1	0.0275	0.7017	0.4299	
BC	0.0266	1	0.0266	0.6773	0.4376	
A^2^	1.43	1	1.43	36.4	0.0005 *	
B^2^	1.11	1	1.11	28.22	0.0011 *	
C^2^	1.55	1	1.55	39.45	0.0004 *	
**Residual**	0.2745	7	0.0392			
Lack of Fit	0.065	3	0.0217	0.4134	0.753	not significant
Pure Error	0.2095	4	0.0524			
**Corrected Total**	7.39	16				
**Response 3: %TR**						
**Source**	**Sum of Squares**	**df**	**Mean Square**	**F-Value**	***p*-Value**	
**Quadratic Model**	103.45	9	11.49	37.64	<0.0001	significant
A-Temperature	2.28	1	2.28	7.45	0.0293 *	
B-Concentration	0.5039	1	0.5039	1.65	0.2398	
C-Solvent	10.06	1	10.06	32.95	0.0007 *	
AB	0.0001	1	0.0001	0.0003	0.9858	
AC	2.94	1	2.94	9.63	0.0173 *	
BC	0.562	1	0.562	1.84	0.217	
A^2^	19.42	1	19.42	63.59	<0.0001 *	
B^2^	12.04	1	12.04	39.44	0.0004 *	
C^2^	47.4	1	47.4	155.22	<0.0001 *	
**Residual**	2.14	7	0.3054			
Lack of Fit	1.22	3	0.4064	1.77	0.2916	not significant
Pure Error	0.9184	4	0.2296			
**Corrected Total**	105.58	16				

df: degree of freedom; *p*-value <0.05 was considered significant; %TF: percentage of theaflavins; %TR: percentage of thearubigins; * variables with a *p*-value < 0.05 are significant.

**Table 5:** Model fit statistical analysis.

	%Extraction Yield	%TF	%TR
**Standard Deviation**	0.9885	0.198	0.5526
**Mean**	24.46	1.09	7.32
**%C.V.**	4.04	18.11	7.54
**R^2^**	0.9891	0.9628	0.9798
**Adjusted R^2^**	0.9751	0.9151	0.9537
**Predicted R^2^**	0.8552	0.815	0.8016
**Adequate Precision**	23.1063	12.0636	18.9109

%TF: percentage of theaflavins; %TR: percentage of thearubigins; %CV: coefficient of variance.

**Table 6:** Second-order polynomial equations and statistical analysis.

	Intercept	A	B	C	AB	AC	BC	A^2^	B^2^	C^2^
**Extraction Yield %**	32.39	−2.52335	1.72478	0.157193	−0.26445	−1.00885	−1.98259	−8.98207	−2.71738	−5.16058
***p*-values**		0.0002 *	0.0017 *	0.6665	0.6092	0.0806	0.0051	<0.0001 *	0.0008 *	<0.0001 *
**%TF**	1.89404	0.007566	−0.07939	0.553254	−0.01167	−0.082941	−0.08149	−0.58226	−0.51262	−0.60614
***p*-values**		0.917	0.2942	<0.0001 *	0.9095	0.4299	0.4376	0.0005 *	0.0011 *	0.0004 *
**%TR**	10.7099	0.533363	−0.25098	1.12156	0.00511	−0.85724	0.374823	−2.14763	−1.69133	3.35529
***p*-values**		0.0293 *	0.2398	0.0007 *	0.9858	0.0173 *	0.217	<0.0001 *	0.0004 *	<0.0001 *

%TF: percentage of theaflavins; %TR: percentage of thearubigins; A: temperature; B: concentration; C: solvent; * variables with a *p*-value < 0.05 are significant.

**Table 7:** Predicted values from response optimization.

	Response Prediction	SE Pred	95% PI
**Extraction Yield %**	32.297	1.08138	29.740–34.855
**%TF**	1.985	0.21663	1.473–2.497
**%TR**	10.771	0.604544	9.341–12.200

SE Pred: Standard Error of Prediction, PI: Prediction interval, %TF: percentage of theaflavins; %TR: percentage of thearubigins.

**Table 8:** Characteristics of empty and black tea transfersomes.

Formulation	MD nm ± SD	PI	ZP mV ± SD	EE % ± SD
**Empty transfersomes (ET)**	61 ± 3.1	0.23 ± 0.01	−40 ± 2.5	--
**Black tea transfersomes (BTT)**	63 ± 5.0	* 0.25 ± 0.02	−40 ± 3.6	Caffeine 86 ± 2.3 Epigallocatechin gallate 93 ± 4.8

Each value represents the mean ± standard deviation (SD; *n* > 10); * *p* < 0.001 *vs*. empty transfersomes; MD: mean diameter; PI: polydispersity index; ZP: zeta potential; EE: entrapment efficiency.

**Table 9:** Characteristics of black tea transfersomes during 150 days at 4 °C.

Black Tea Transfersomes	MD nm ± SD	PI	ZP mV ± SD
**t_0_**	63 ± 5.0	0.25 ± 0.02	−40 ± 3.6
**t_15_**	63 ± 5.2	0.22 ± 0.01	−43 ± 1.9
**t_30_**	63 ± 5.4	0.23 ± 0.01	−43 ± 2.2
**t_60_**	66 ± 2.7	0.26 ± 0.04	−46 ± 2.2
**t_90_**	66 ± 1.8	0.25 ± 0.01	−41 ± 5.4
**t_120_**	66 ± 4.6	0.26 ± 0.01	−42 ± 6.2
**t_150_**	71 ± 8.7	0.30 ± 0.07	−42 ± 6.5

Each value represents the mean ± standard deviation (SD; *n* > 10); MD: mean diameter; PI: polydispersity index; ZP: zeta potential.

## Data Availability

The original contributions presented in this study are included in the article. Further inquiries can be directed to the corresponding author.
